# Chimeric Murine Polyomavirus Virus-Like Particles Induce *Plasmodium* Antigen-Specific CD8^+^ T Cell and Antibody Responses

**DOI:** 10.3389/fcimb.2019.00215

**Published:** 2019-06-19

**Authors:** David J. Pattinson, Simon H. Apte, Nani Wibowo, Yap P. Chuan, Tania Rivera-Hernandez, Penny L. Groves, Linda H. Lua, Anton P. J. Middelberg, Denise L. Doolan

**Affiliations:** ^1^Infectious Diseases Programme, QIMR Berghofer Medical Research Institute, Brisbane, QLD, Australia; ^2^Centre for Molecular Therapeutics, Australian Institute of Tropical Health and Medicine, James Cook University, Cairns, QLD, Australia; ^3^Australian Institute for Bioengineering and Nanotechnology, University of Queensland, Brisbane, QLD, Australia; ^4^Protein Expression Facility, University of Queensland, Brisbane, QLD, Australia

**Keywords:** malaria, vaccine, circumsporozoite protein, virus-like particle, murine polyomavirus, cellular immunity, T cell responses, *Plasmodium yoelii*

## Abstract

An effective vaccine against the *Plasmodium* parasite is likely to require the induction of robust antibody and T cell responses. Chimeric virus-like particles are an effective vaccine platform for induction of antibody responses, but their capacity to induce robust cellular responses and cell-mediated protection against pathogen challenge has not been established. To evaluate this, we produced chimeric constructs using the murine polyomavirus structural protein with surface-exposed CD8^+^ or CD4^+^ T cell or B cell repeat epitopes derived from the *Plasmodium yoelii* circumsporozoite protein, and assessed immunogenicity and protective capacity in a murine model. Robust CD8^+^ T cell responses were induced by immunization with the chimeric CD8^+^ T cell epitope virus-like particles, however CD4^+^ T cell responses were very low. The B cell chimeric construct induced robust antibody responses but there was no apparent synergy when T cell and B cell constructs were administered as a pool. A heterologous prime/boost regimen using plasmid DNA priming followed by a VLP boost was more effective than homologous VLP immunization for cellular immunity and protection. These data show that chimeric murine polyomavirus virus-like particles are a good platform for induction of CD8^+^ T cell responses as well as antibody responses.

## Introduction

Although annual malaria related mortality rates have decreased by approximately 25% since 2010, improvements in morbidity and mortality have stabilized in recent years (World Malaria Report, 2017) and rebounds have been reported in some countries (Alonso et al., 2011). This is despite an annual financial investment of approximately US$2.7 billion, which includes malarial preventative measures such as residual spraying, insecticide-treated mosquito nets and preventative therapies (World Malaria Report, 2017). An effective vaccine is considered by many to be an essential tool for malaria disease control and eradication (Alonso et al., 2011). The ideal vaccine would target the pre-erythrocytic stage to induce sterile infection-blocking immunity which either prevents sporozoite invasion of the hepatocyte and/or halts the development of the parasite at the liver-stage; this would stop the development of clinical symptoms of malaria which manifest during the blood-stage, as well as the transmission of malaria which occurs during the sexual stage. A partially effective vaccine which reduced liver-stage parasite burden and thus lowered blood-stage parasitemia to potentially below a threshold associated with clinical disease would, however, also be a useful tool.

Recently, GlaxoSmithKline achieved a significant milestone with Mosquirix^TM^ (also known as RTS,S) by receiving a positive scientific opinion from the European Medicines Agency as the first malaria vaccine for the immunization of children aged 6 weeks to 17 months. This sub-unit virus-like particle (VLP) based vaccine targets the *P. falciparum* circumsporozoite protein (*Pf* CSP) by combining a protein which includes multiple *Pf* CSP B cell repeats and T cell epitopes fused with recombinant hepatitis B surface antigen (RTS), and recombinant wild-type hepatitis B surface antigen (S) (Gordon et al., 1995). These recombinant proteins combine to form stable VLPs, which are co-administered with the AS01 adjuvant (Agnandji et al., 2012). Disappointingly, clinical studies have shown that although RTS,S provided some protection in the first year after vaccination, this efficacy was very low and waned quickly (Tinto et al., 2015; Olotu et al., 2016) with negative efficacy and a rebound in later years demonstrated in a long-term follow-up study where RTS,S immunized 5–17 month children were more likely to be infected 5 years following vaccination (Olotu et al., 2016). These data emphasize the urgent need to identify new vaccine targets and vaccine delivery platforms that enhance immunogenicity and provide durable protection.

The CSP remains the most advanced subunit vaccine candidate, as a target for neutralizing antibodies prior to liver infection (Charoenvit et al., 1991; Mishra et al., 2012) and for T cell responses whilst in the hepatocyte (Grillot et al., 1990; Weiss et al., 1990, 1992; Franke et al., 1997, 2000). The induction of T cell or antibody mediated protection against sporozoite challenge has been demonstrated in murine and non-human primate models as well as humans by various vaccine platforms based on the CSP (Sedegah et al., 1994; Walsh et al., 2006; Mettens et al., 2008; Tewari et al., 2010; Tamminga et al., 2011; Noe et al., 2014; Janitzek et al., 2016; Collins et al., 2017; Yoshida et al., 2018). Thus, the CSP is widely considered the antigen of choice for evaluating novel vaccine delivery platforms targeting the pre-erythrocytic stage of the parasite life cycle.

Whilst antibody responses generated by chimeric VLP immunizations using various VLP platforms with antigens from various pathogens in clinical studies are well-documented (reviewed in Kushnir et al., 2012), little research has been done to evaluate their efficacy at generating cellular responses. Chimeric VLPs using hamster and simian virus 40 polyomavirus structural proteins have shown antigen-specific cytotoxic T lymphocyte (CTL) responses with viral epitopes (Mazeike et al., 2012; Kawano et al., 2014), and a murine polyomavirus VLP with large HER-2_1−683_ oncogene insert mouse study showed rejection and inhibition of antigen expressing tumor cells *in vivo* (Tegerstedt et al., 2005). Furthermore, both HPV16 L1 VLPs and their subunit component capsomeres are potent inducers of CTL responses capable of tumor regression without adjuvants (Ohlschläger et al., 2003).

In this study, we produced chimeric VLPs using the murine polyomavirus (MuPyV) (Salunke et al., 1986) by genomic insertion of either *Plasmodium yoelii* CSP $\text{CD}8_{\left(280-288\right)}^{+}$ (Weiss et al., 1992) or $\text{CD}4_{\left(59-79\right)}^{+}$ (Grillot et al., 1990) T cell epitopes, or the B cell repeat epitope_(QGPGAPx2)_ into a surface-exposed region of the VP1 structural protein (Middelberg et al., 2011). We selected the MuPyV-VP1 platform which has been extensively developed by our group, and can be produced in bacterial expression systems which can be purified at gram per liter levels (Liew et al., 2010). The proteins form pentameric capsomeres (Salunke et al., 1986) which can be chemically induced *in vitro* to self-assemble into VLPs (Chuan et al., 2008a; Middelberg et al., 2011; Rivera-Hernandez et al., 2013). We comprehensively evaluated antibody and T cell responses as well as protection from *Plasmodium yoelii* 17XNL sporozoite challenge using chimeric *Py*CSP surface exposed on murine polyomavirus VLP constructs delivered separately or pooled.

Consistent with previous results with the murine polyomavirus VLPs (Anggraeni et al., 2013; Rivera-Hernandez et al., 2013; Wibowo et al., 2014; Seth et al., 2016; Tekewe et al., 2017), the *Plasmodium* B cell VLP was a strong inducer of antigen-specific antibody responses. Additionally, we showed that the VLP platform was capable of inducing robust VLP-induced CD8^+^ T cell responses. However, the VLPs did not stimulate a good CD4^+^ T cell response, and there was no apparent help provided by the CD4 VLP to the VLP-induced CD8^+^ T cell responses nor to the induced antibody titers. The VLP-induced CD8^+^ T cell responses were however enhanced using a heterologous DNA prime-boost regimen.

## Materials and Methods

### Plasmid Construction

The plasmid pGEX-4T-1 (GE Healthcare Biosciences, UK) containing the murine polyomavirus VP1 sequence (M34958) was provided by Professor Robert Garcea (University of Colorado, USA). The VP1 sequence was modified by inserting an *Afe*I restriction enzyme site flanked with Glycine_4_-Serine linker sequences at position 293 and designated pGEX-VP1-S4-G4S (Middelberg et al., 2011). This allows for antigens to be inserted into a surface-exposed region of the VP1 protein with the linker sequences added to reduce structural interference from the insert on VLP formation. Codon optimized sequences representing defined *Py*CSP CD8^+^_(280−288)_ (Weiss et al., 1992) and CD4^+^_(59−79)_ (Grillot et al., 1990) T cell epitopes and the B cell repeat epitope _(QGPGAPx2)_ (Charoenvit et al., 1991) were separately inserted into the *Afe*I site in pGEX-VP1-S4-G4S using standard molecular biology techniques to generate chimeric CD8^+^ or CD4^+^ T cell or B cell VLP constructs. All constructs were confirmed by Sanger sequencing.

### Protein Expression, Purification, and VLP Assembly

Wild-type pGEX-VP1-S4-G4S, or the chimeric CD8^+^, CD4^+^, or B cell constructs were individually transformed into chemically competent *E. coli* Rosetta DE3 pLysS bacteria (Novagen, CA, USA). The GST-tagged VP1 proteins were expressed and purified as previously described (Chuan et al., 2008a; Middelberg et al., 2011). Briefly, bacteria inoculated Terrific Broth was incubated at 37°C and 180 RPM until the OD_600_ reached approximately 0.5, after which the culture was cooled to 26°C and induced by adding 0.2 mM Isopropyl β-D-1-thiogalactopyranoside (IPTG) and incubated overnight at 26°C and 180 RPM. The cultures were centrifuged and pellets lysed by sonication then filtered lysate was passed through a GSTrap HP affinity column (GE Healthcare, UK) and the purified protein was cleaved from the GST tag using thrombin (GE Healthcare, UK) and polished using a Superdex 200 10/300 GL column (GE Healthcare, UK). Endotoxin levels were reduced (<5 EU/ml) using Vivapure Q maxi H ion exchange columns (Sartorius Stedim, Germany) and confirmed with an Endosafe PTS reader (Charles River Laboratory, USA) as previously described (Middelberg et al., 2011). VP1 capsomeres were assembled into VLPs by dialysis against an assembly buffer then against PBS (Chuan et al., 2008a; Liew et al., 2010; Middelberg et al., 2011). The resultant VLPs were analyzed by asymmetric flow field-flow fractionation coupled to multi angle light scattering (AF4-MALS) using an Eclipse 2 AFFFF system coupled with a Dawn EOS MALS system (Wyatt Technology Corporation, Santa Barbara, USA), and transmission electron microscopy (TEM) using a JEOL 1010 (JEOL Ltd., Tokyo, Japan) to assess size distribution as previously described (Chuan et al., 2008b; Lipin et al., 2008).

### Immunization of Mice

**Figure 1:**
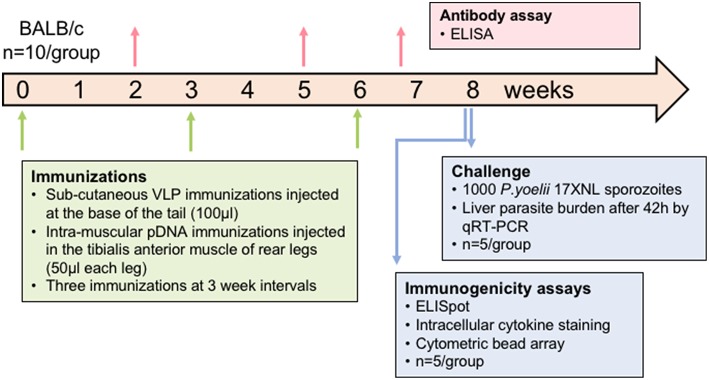
Schematic of experimental design. In each experiment, mice (*n* = 10/group) were immunized three times at a 3-week interval. Plasmid DNA injections were intramuscular into the tibialis anterior whilst VLP immunizations were subcutaneous on the back near the base of the tail. Blood samples were collected 14 days after doses 1 and 2 and then 5 days after the final dose to assess antibody responses. Half of the mice were euthanized at 10 days after the final dose and splenocytes used for immunogenicity assays. The other half were challenged 2 weeks after the final dose by injecting 1,000 *P. yoelii* sporozoites into a tail vein, and livers removed 42 h later to assess parasite load by qRT-PCR. In one experiment, the VLP doses were 30 μg for individual VLPs or 10 μg of each in the pooled VLP group. In another experiment, the pooled VLP dose was increased to 30 μg of each VLP and a DNA-only control was included.

All animal experiments were approved by the QIMR Berghofer Animal Ethics Committee and were conducted in accordance with the Australian Code of Practice for the Care and Use of Animals for Scientific Purposes (2004). BALB/c mice (*n* = 10/group) aged 7–8 weeks (Animal Resources Center, WA, Australia) were immunized with the chimeric VLP constructs three times at 3-week intervals by subcutaneous injection at the base of the tail. VLPs were administered at two doses, 10 or 30 μg in independent experiments, and individually or pooled. VLPs were administered without adjuvant as this platform is considered self-adjuvating (Chackerian et al., 2002; Stanley, 2006). Synthetic peptides corresponding to the epitopes presented by the chimeric VLP co-administered with 50 μg of high molecular weight poly (I:C) adjuvant (Invivogen, USA) were included as comparator groups in each experiment. In heterologous prime-boost regimens, mice received two doses of 100 μg of *Py*CSP plasmid DNA (pVR2516, Vical Inc, CA, USA) at 3-week intervals by intramuscular injection into the tibialis anterior muscle, followed by a booster dose of pooled VLP or synthetic peptide in poly(I:C) adjuvant as previously described. In the second experiment, one group received three homologous immunizations with *Py*CSP plasmid DNA at 100 μg per dose. Control groups included wild-type VLPs, ovalbumin $\text{CD}8_{257-264}^{+}$ and $\text{CD}4_{323-339}^{+}$ pooled peptides with 50 μg poly(I:C), and PBS only (naïve). Immunized mice were split into two groups (*n* = 5/group) to evaluate either immunogenicity using splenocytes for T cell assays or protective efficacy by sporozoite challenge (Figure 1).

### Sporozoite Challenge and Protection

Two weeks after the final immunization, mice were challenged with 1,000 cryopreserved *P. yoelii* 17 XNL sporozoites (Sanaria Inc., MD, USA) in 200 μl PBS with 2% naive mouse serum administered intravenously into the tail vein. Livers were harvested 42 h post-challenge to assess liver-stage parasite burden as previously described (Schussek et al., 2013). Briefly, livers were homogenized in 5 ml of RLT buffer (Qiagen, Netherlands) supplemented with 1% β-2-mercaptoethanol (Sigma-Aldrich), then RNA was extracted using an RNeasy Mini kit (Qiagen) following the manufacturer's protocol. Using 2.5 μg of RNA, cDNA was synthesized using a Super Script VILO reverse-transcriptase cDNA synthesis kit (Life Technologies, USA) according to manufacturer's guidelines. Then the *P. yoelii* 18S rRNA and murine housekeeping gene glyceraldehyde 3-phosphate dehydrogenase (GAPDH) cDNA were quantified using quantitative reverse transcriptase-PCR (qRT-PCR) using a Rotor-gene 3000 (Corbett Research, Mortlake, Australia) with acquisition on the FAM channel. For *Py*18S cDNA quantification, a Taqman Fast Advanced master mix (Applied Biosystems, Australia) with a custom made Taqman probe [250 nm] (6FAM-CTGGCCCTTTGAGAGCCCACTGATT-BHQ-1) and primers [1 um] (5″- CTTGGCTCCGCCTCGATAT and 3″- TCAAAGTAACGAGAGCCCAATG) (Applied Biosystems). A GAPDH kit (Applied Biosystems) combined with platinum Taq polymerase, PCR buffers (Invitrogen) was used for GAPDH quantification. The data are presented as a ratio of copies of *Py*18S rRNA per 10^5^ copies of GAPDH.

### Splenocyte Harvesting and Stimulation for T Cell Assays

Ten days after the final immunization, spleens were removed and single cell suspensions were generated by mechanical disruption followed by red blood cell lysis. Splenocytes were then co-incubated with gamma irradiated (16,666 cGy) mouse B cell lymphoma A20 cells (ATCC TIB-208) which were transfected, peptide stimulated, or untreated. Transfections with *Py*CSP encoded plasmid DNA pVR2516 (Vical, USA) or empty vector pVR1020 (Vical, USA) plasmid DNA was achieved the AMAXA Nucleofector system (Lonza, Switzerland) using Kit V and program C-25 with 5 x 10^6^ A20 cells per cuvette, following the manufacturer's protocol. Peptide stimulation used either *Py*CSP CD8^+^_(280−288)_ or CD4^+^_(59−79)_ T cell epitope synthesized peptides, or these peptides combined with the B cell repeat epitope (QGPGAPQGPGAP) peptide at 10 μg/ml. For this assay, cells were incubated in KD-MEM media comprised of Dulbecco's Modified Eagle's Medium (SAFC Global, USA) supplemented with folic acid (136 nM), L-asparagine (32 mM), L-arginine (67 mM), sodium bicarbonate (24 mM), HEPES (10 mM), β-2-mercaptoethanol (5 nM), L-glutamine (1.5 mM), penicillin (100 Units/L), streptomycin (100 mg/L) and 10% fetal calf serum. Each well-contained 5 x 10^5^ splenocytes and 1.5 × 10^5^ A20 cells in 200 μl of media. Background responses were removed by subtracting transfected pVR1020 wells from pVR2516 wells, and untreated A20 wells from peptide-pulse wells for ELISpot, cytometric bead array (CBA) and intracellular cytokine staining (ICS) assays.

### IFN-γ ELISpot Assay

IFN-γ ELISpot assays were conducted as previously described (Schussek et al., 2017). Briefly, MSIPS4510 multiscreen ELISpot plate (Merck Millipore, Germany) well were pre-coated with 100 μg/ml anti-mouse IFN-γ (BD Biosciences, USA) then blocked with KD-MEM supplemented with 10% FCS. Quadruplicate wells were used for each stimulation. Plates were incubated for 40 h at 37°C and 5% CO_2_. After plate washing, IFN-γ secreting cells were stained with 2 μg/ml biotinylated anti-mouse IFN-γ (BD Biosciences, USA) followed by 1 μg/ml streptavidin-HRP (BD Biosciences, USA). The assay was developed using AEC substrate (BD Biosciences, USA), and spots counted using the AID ELISpot reader system (Autoimmun Diagnostika GmbH, Germany).

### Cytometric Bead Array

Splenocyte/A20 cultures were incubated at 37°C and 5% CO_2_ for 72 h. Culture supernatant was collected and secreted IFN-γ, TNF, IL-1β, IL-2, IL-4, IL-5, IL-6, IL-10, IL-12p70, and IL-13 cytokines assayed using the mouse cytometric bead array flex kit (BD Biosciences, USA) following the manufacturer's protocol. Samples were analyzed using a FACSArray instrument (BD Biosciences, USA) using the CBA array software (BD Biosciences, USA).

### Intracellular Cytokine Staining

To detect monofunctional or polyfunctional T cell responses, splenocyte/A20 cultures were incubated with 0.1% Golgi Plug (BD Biosciences, USA) for 6 h at 37°C and 5% CO_2_. Cells were stained with anti-CD8^+^ (53-6.7) and anti-CD4^+^ (RM4.5) antibodies before being fixed with 1% paraformaldehyde. Cells were then stained with anti-IFN-γ (XMG1.2), anti-IL-2 (JES6-5H4), and anti-TNF-α (MP6-XT22) diluted in permwash buffer (BD Biosciences, USA). All antibodies were purchased from Biolegend. Flow cytometric analysis was performed on a Fortessa 4 (BD Biosciences, USA). Post-acquisition data analysis was performed using FlowJo software version 10 (Treestar, USA).

### ELISA

Antigen-specific antibody responses were detected by ELISA using the *Py*CSP B cell repeat peptide linked to a polystyrene binding tag (Kumada et al., 2010) with a glycine_4_ spacer (Kogot et al., 2012) (PST-B cell, RIIIRRIRGGGG-QGPGAPx3) (Mimotopes, Australia). The PST-tag with a glycine spacer was incorporated to enhance attachment of the peptide to the plate and expose the B cell repeat for antibody recognition. Nunc maxisorp plates (Thermo Fisher Scientific, USA) were coated overnight with the PST-B cell peptide (5 μg/ml) in a carbonate coating buffer, then blocked with PBS containing 2% BSA. Triplicate wells of sera from mice were titrated by 2-fold dilutions in PBS-BSA 0.1%, and then incubated with biotinylated donkey α-mouse IgG (Jackson ImmunoResearch Laboratories, USA) diluted in PBS-BSA 0.1%, followed by an incubation with streptavidin-HRP (BD Biosciences, USA) diluted 1:1,000 in PBS-BSA 0.1 and 0.2% Tween20. Plates were developed with tetramethylbenzidine (TMB) and stopped using TMB stop reagent (Sigma Aldrich, USA). Absorbance was measured at 450 nm using a VersaMax microplate reader (Molecular Devices, USA). Positive results were recorded if OD_450_ values were > 3 standard deviations above the mean blank (no serum) values.

### Statistical Analysis

Statistical analysis was performed using GraphPad Prism version 6.0 (GraphPad, CA, USA). Logarithmic transformed data of groups were compared by one-way analysis of variance (ANOVA) and Bonferroni's multiple comparison test. Statistical significance is displayed as ^*^p < 0.05, ^**^p < 0.01, ^***^p < 0.001, and ^****^p < 0.0001.

## Results

### VLP Construction

**Figure 2:**
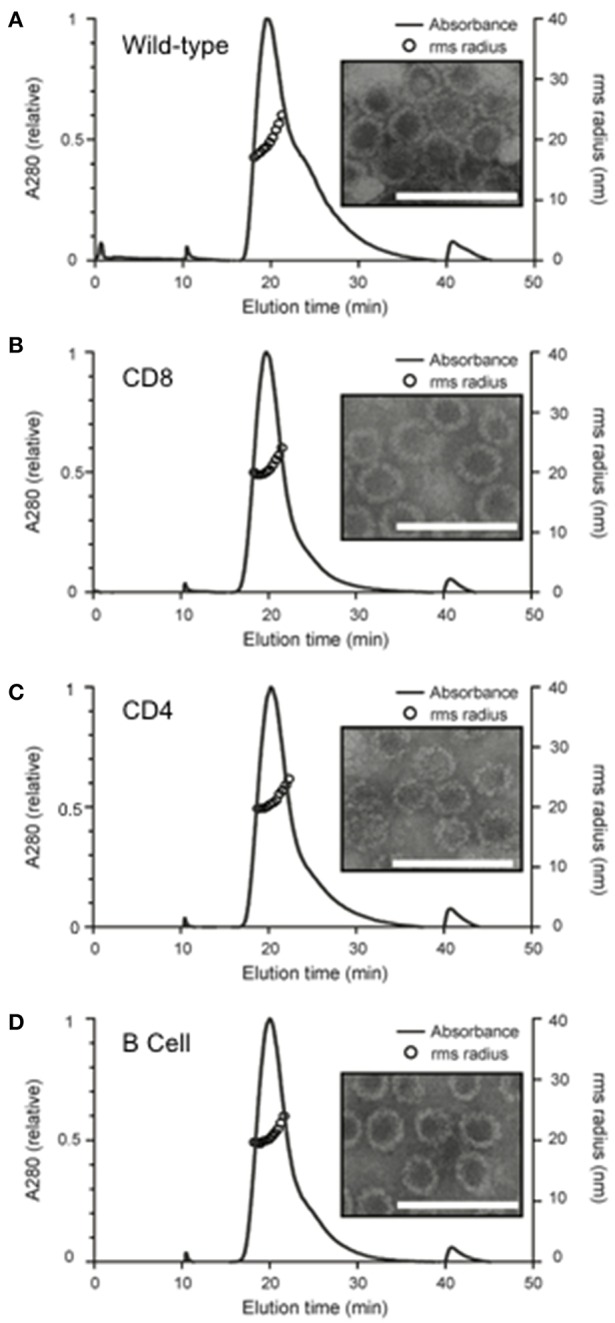
Structural analysis of *in vitro* assembled chimeric murine polyomavirus virus-like particles. Wild and chimeric MuPyVP1-G4S proteins were expressed in *E. coli* and purified by liquid chromatography. Proteins were dialyzed against an assembly buffer to form VLPs and then against PBS. Post-assembly solutions were analyzed to detect the formation of VLPs using asymmetrical flow field- flow fractionation coupled with multi-angled lights scattering (AF4-MALS) and transmission electron microscopy of **(A)** wild-type VLPs, **(B)** CD8 VLP chimeras, **(C)** CD4 VLP chimeras, and **(D)** B cell VLP chimeras. UV_280_ absorbance is displayed relative to peak absorbance (solid line) for each sample, and particle size is shown as root-square-radius (open circles). Scale bar is 100 nm.

In-frame genomic insertion of antigen-derived epitopes into the MuPyV-VP1 protein was confirmed by sequencing. After the VLPs were assembled *in vitro*, analysis using AF4-MALS showed only small amounts of protein aggregation and TEM images confirmed that the chimeras had a similar morphology to wild-type VLPs (Figure 2). The mean radius of each of the chimeric VLPs was similar to that of the wild type VLPs [Wild type- 21.00 ± 1.27 nm; CD8 VLPs- 20.51 ± 0.67 nm; CD4 VLPs- 21.07 ± 0.61 nm; B cell VLPs- 20.85 ± 0.67 nm (mean radius ± SD)].

### VLP-Induced T Cell Immune Responses

Mice were immunized with chimeric CD8 or CD4 VLPs to assess their capacity independently to induce CD8^+^ or CD4^+^ T cell responses, and to evaluate if pooling of the VLPs would result in an additive or synergistic response. Pooled VLPs were evaluated in independent experiments at 10 μg per dose and at 30 μg per dose to dose-match the independent VLPs (Figure 1).

**Figure 3:**
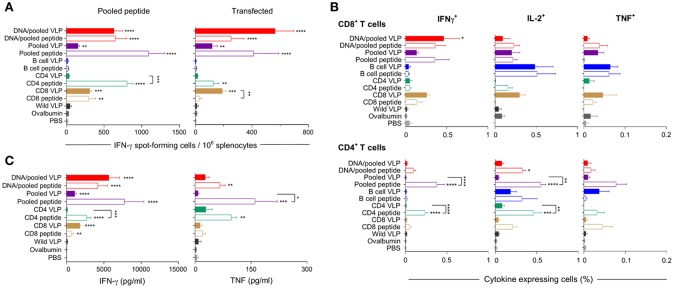
Cellular immune responses induced by individual (30 μg) or pooled (10 μg each) chimeric VLP immunizations. BALB/c mice (*n* = 5/group) were immunized with a prime/boost regimen using two i.m., priming immunizations with *Py*CSP plasmid DNA (100 μg) followed by a single s.c. boost using either pooled *Py*CSP VLPs (10 μg each) or pooled *Py*CSP peptides (30 μg each) with poly(I:C); or with three homologous s.c doses of pooled *Py*CSP VLPs (10 ug each) or individual *Py*CSP VLPs (30 ug); or pooled or individual *Py*CSP peptides (30 μg each) with poly(I:C); wild type VLPs (30 μg); pooled ovalbumin peptides (30 μg each) with poly(I:C); or PBS only. Ten days after the final boost, splenocytes (5 × 10^5^/well) were cultured with irradiated A20 cells pulsed with pooled *Py*CSP CD8_280−288_, CD4_59−79_, or B cell peptides (1.5 × 10^5^/well), or with irradiated A20 cells transfected with pVR2516 *Py*CSP plasmid DNA (5 × 10^4^/well), for assessment of T cell specific immune responses. **(A)** IFN-γ ELISpot was used to determine IFN-γ spot forming cells/million splenocytes following 40 h incubation with pooled CD8_280−288_ plus CD4_59−79_ peptides (left) or DNA transfected A20 cells (right). **(B)** ICS was used to determine CD8^+^ and CD4^+^ T cell, IFN-γ, IL-2, and TNF cytokine responses following 18 h stimulation with pooled peptides, with the final 6 h supplemented with GolgiPlug. **(C)** Cytometric Bead Array (CBA) was used to quantify cytokine concentrations in 72 h culture supernatant following pooled peptide stimulation. All data are presented as group means + SEM (media only subtracted). Statistical comparisons are made to the PBS control group, and between homologous VLP and peptide immunization groups using log-transformed data with significance determined using one-way ANOVA followed by Bonferroni's *post-hoc* test. *p < 0.05, **p < 0.01, ***p < 0.001, and ****p < 0.0001.

**Figure 4:**
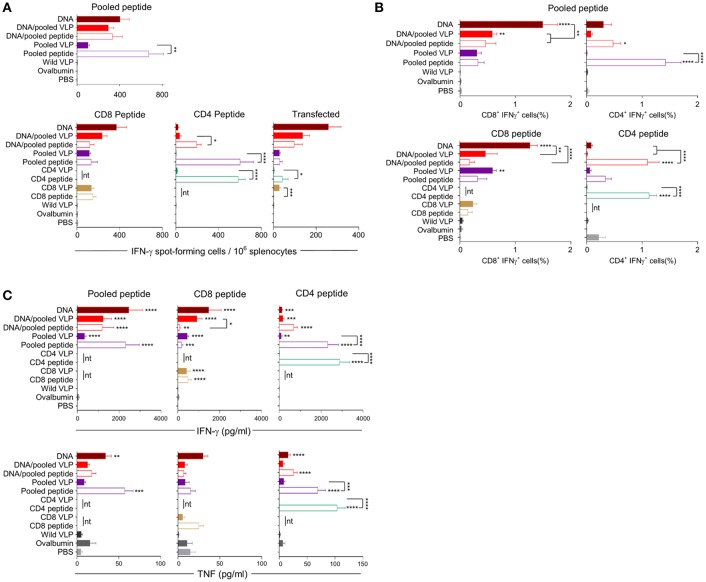
Cellular immune responses induced by individual (30 μg) or pooled (30 μg each) chimeric VLP immunizations. BALB/c mice (*n* = 5/group) received three immunizations at 3 week intervals with three homologous i.m., *Py*CSP plasmid DNA (100 μg) immunizations or a prime/boost regimen using two i.m., priming immunizations with *Py*CSP plasmid DNA (100 μg) followed by a single s.c. boost using either pooled *Py*CSP VLPs (30 μg each) or pooled *Py*CSP peptides (30 μg each) with poly(I:C); or three homologous s.c doses of pooled or individual *Py*CSP VLPs (30 μg each) or *Py*CSP peptides (30 μg each) with poly(I:C); wild type VLPs (30 μg); pooled ovalbumin peptides (30 μg each) with poly(I:C); or PBS only. **(A)** IFN-γ ELIspot, **(B)** ICS, and **(C)** CBA responses were assayed as described in the legend to Figure 3, using individual CD8 peptides or CD4 peptides, pooled peptides, or DNA-transfected A20 cells. All data sets are presented as group means + SEM (media only subtracted). Statistical comparisons are made to the PBS control group, and between homologous VLP and peptide immunization groups using log-transformed data with significance determined using one-way ANOVA followed by Bonferroni's *post-hoc* test. *p < 0.05, **p < 0.01, ***p < 0.001, and ****p < 0.0001 (nt, not tested).

IFN-γ responses detected by ELISpot showed that homologous immunization with the CD8 VLP induced significant IFN-γ spot forming cells (SFCs) when stimulated with pooled peptide or *Py*CSP transfected A20 cells (*p* < 0.001) (Figures 3A, 4A). The magnitude of VLP-induced CD8^+^ T cell response was similar to that induced by CD8 peptide immunization when stimulated by peptide (but not transfected A20 cells). In contrast, the CD4 VLP failed to induce a significant IFN-γ response (Figures 3A, 4A); robust responses against peptide (*p* < 0.0001) and transfected stimulation (*p* < 0.001) were observed in mice immunized in parallel with CD4 peptides in adjuvant (Figure 3A) and similarly in the subsequent experiment (Figure 4A).

Immunization with pooled CD8 plus CD4 VLPs resulted in a similar IFN-γ profile to that induced by the individual VLPs, with a robust response to the CD8 peptide and negligible response to the CD4 peptide (Figure 4A).

Heterologous prime-boost immunization with DNA prime/VLP boost induced a stronger response than homologous VLP immunization (Figures 3A–C). This was attributed to the plasmid DNA prime with the homologous DNA regimen inducing the most robust response (Figures 4A–C). The reduction in responses between the DNA only and DNA prime/VLP boost could be due to an inhibition caused by VLPs, but more likely that the third dose of DNA was more immunogenic than the VLPs.

The profile of IFN-γ responses detected with both CBA and ICS readouts was similar to that shown by ELIspot for the CD8 and CD4 VLPs (Figures 3A–C, 4A–C). Robust IFN-γ responses induced by the CD8 VLP were similar to that induced by peptide in adjuvant. Negligible CD4^+^ T cell responses were induced by CD4 VLP immunizations, as opposed to the robust CD4^+^ T cell responses which were evident following CD4 peptide immunization. This differentiation is likely due to the reduced actual peptide load in the CD4 VLPs of approximately 0.52 μg.

TNF was induced in mice immunized with the CD4 peptide individually or in pools (Figures 3C, 4C). TNF was also significantly induced by DNA immunizations in response to pooled peptide (*p* < 0.01) and CD4 peptide stimulation (*p* < 0.0001). No VLP immunizations resulted in significant TNF or IL-2 production from stimulated splenocytes. Our CBA assessed a range of cytokines specifically: IFN-γ, L-1β, IL-2, IL-4, IL-5, IL-6, IL-10, IL-12p70, IL-13 and TNF. However, with the exception of IFN-γ and TNF, we only detected a very low level of cytokines induced by our VLP immunizations (data not shown).

### VLP-Induced Antibody Responses

The B cell VLP was predicted to induce strong antibody responses since it comprised the dominant *Py*CSP B cell linear epitope exposed on the surface of the VLP in a repetitive array. Responses to the B cell VLP were assessed in parallel individually as well as in a pool with the CD8 and CD4 VLPs since it was anticipated that the inclusion of the CD4 VLP would enhance the epitope-specific antibody titer.

**Figure 5:**
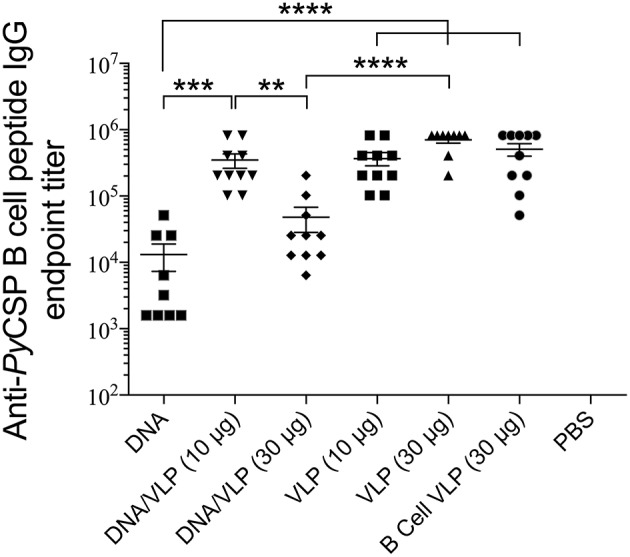
Anti-*Py*CSP B cell peptide IgG responses induced by immunization with individual or pooled VLP immunizations. In separate series of experiments, mice (*n* = 10/group) were immunized with individual VLPs (30 μg/dose) or pooled VLPs (either 10 μg/construct or 30 μg/construct), as described in the legends to Figures 3, 4. Sera collected 5 days following the final immunization were batch analyzed by ELISA to determine the anti-B cell peptide-specific IgG endpoint titer. Data is presented as mean endpoint titer + SEM. Statistical comparisons between immunization groups used log-transformed data with significance determined using one-way ANOVA followed by Bonferroni's *post-hoc* test. **p < 0.01, ***p < 0.001, and ****p < 0.0001.

Robust anti-*Py*CSP B cell peptide IgG responses were induced by all groups containing the B cell VLP (*p* < 0.0001 relative to naive) (Figure 5). In the homologous VLP immunizations, there were no significant differences between any groups indicating no additive effect by including the CD4 VLP. VLP-induced antibody responses were significantly higher than homologous DNA immunizations (*p* < 0.0001). In the DNA prime/VLP boost regimen, the response induced by the 10 μg/construct boost was significant higher as compared to the 30 μg/construct boost (*p* < 0.01); conversely in the homologous VLP only groups, the 30 μg per dose group higher than the 10 μg per dose, although not significantly different.

### Protection From Sporozoite Challenge

**Figure 6:**
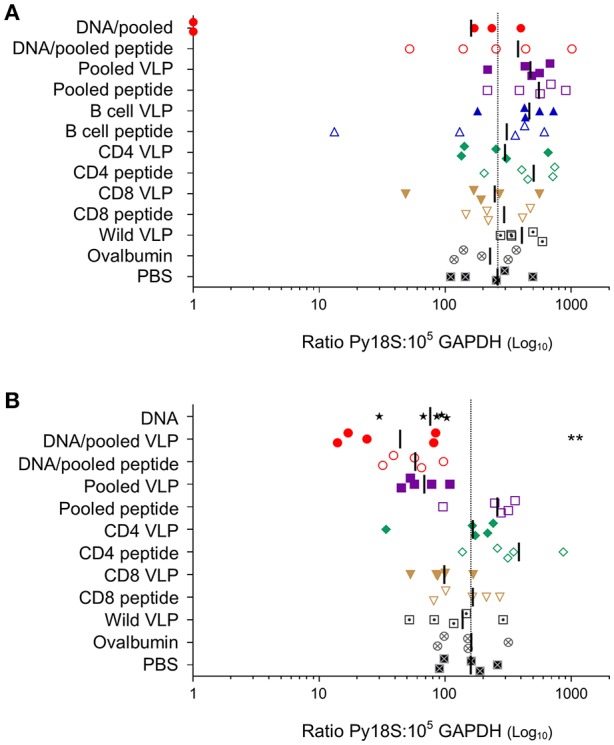
Protective capacity induced by individual or pooled chimeric VLPs using homologous or heterologous immunization regimens. In two independent experiments **(A)** BALB/c mice (*n* = 5/group) were immunized with three immunizations at 3-week intervals (as in Figure 3) with a prime-boost regimen using two i.m. *Py*CSP plasmid DNA (100 μg) primes followed by a s.c. boost of either pooled *Py*CSP VLPs (10 μg each) or pooled *Py*CSP peptides (30 μg each); or three homologous s.c. immunizations with pooled *Py*CSP VLPs (10 μg each) or pooled PyCSP peptides (30 μg each) in poly(I:C); or individual *Py*CSP B cell, CD4 or CD8 VLPs (30 μg) or corresponding peptides (30 μg) with poly(I:C); or wild-type VLPs (30 μg); or ovalbumin pooled peptides (30 μg each) with poly(I:C); or PBS only. (B) BALB/c mice (*n* = 5/group) were immunized with three immunizations at 3-week intervals (as in Figure 4) with three i.m immunizations of *Py*CSP plasmid DNA (100 μg); a prime/boost regimen or homologous regimens as above with dosing modifications to pooled *Py*CSP VLPs increasing to 30 μg per VLP. In both experiments, mice were challenged 2 weeks after the final immunization by i.v., injection of 1,000 *P. yoelii* 17XNL cryopreserved sporozoites. Parasite burden was assessed by qRT-PCR analysis of *Py*18s rRNA extracted from livers harvested 42 h post-challenge and calculated as a ratio to the GAPDH housekeeping-gene mRNA. Results for individual mice are shown, with bars representing the group mean. The dotted line represents the naïve mouse mean. Log-transformed ratio data was analyzed using one-way ANOVA followed by Bonferroni's *post-hoc* test. **p < 0.01.

To evaluate the protective efficacy of chimeric VLPs, VLP-immunized mice (*n* = 5/group) were challenged with 1,000 *P. yoelii* 17XNL sporozoites. In the first experiment, 40% (2/5) mice immunized with DNA prime / VLP boost had no parasite RNA detected in their liver and there was a 38% reduction in liver-stage parasite burden relative to the PBS control group overall in the group; these differences were not significant given the relatively small group sizes. When the experiment was repeated using the higher dose pooled VLPs (30 μg/construct), a significant reduction in parasite burden was noted in mice immunized with the heterologous DNA prime / VLP pooled VLP regimen (*p* < 0.01) (Figure 6). This equates to a mean 72% reduction in the liver parasite burden relative to the PBS immunized group. In the pooled VLP immunized mice, there was a mean 57% reduction in liver-stage parasite burden relative to the PBS control, although this did not reach statistical significance.

## Discussion

Development of an efficacious malaria vaccine has been challenging, with even the most advanced candidate Mosquirix^TM^ showing very poor efficacy despite very extensive refinement during preclinical development and early stage clinical testing (Tinto et al., 2015; Olotu et al., 2016). Mosquirix™ is a chimeric VLP construct expressing the central repeat region plus the entire C-terminal flanking region (amino acids 207–395) of the *P. falciparum* CSP fused to the hepatitis B virus surface antigen, HBsAg. Protection has been associated with antibodies or CD4^+^ T cell responses, but this vaccine fails to induce CD8^+^ T cell responses which are thought to be required for protection against intracellular pathogens such as the *Plasmodium* spp. parasite (Seder and Hill, 2000). Various *Plasmodium* targets have been evaluated using a range of subunit vaccine delivery platforms including recombinant protein, recombinant viruses, synthetic peptides, plasmid DNA, nanoparticles, and VLPs [reviewed in Draper et al. (2015)]. However, none of these platforms have induced robust CD8^+^ and CD4^+^ T cell responses as well as antibody responses sufficient for protection against malaria. In other disease models, VLP vaccines show potential for induction of broad antibody and cellular immune responses.

Protection directed against the pre-erythrocytic stage of the *Plasmodium* lifecycle following sporozoite challenge can be achieved primarily by antibody responses which inhibit sporozoite infection of the hepatocyte (Majarian et al., 1984; Charoenvit et al., 1991) or CD8^+^ T cell responses which act directly or indirectly upon the liver-stage parasite (Weiss et al., 1988, 1990, 1992; Doolan and Hoffman, 2000); moreover, there is evidence that induction of CD4^+^ T cell responses is required for optimal efficacy (Weiss et al., 1993). There is increasing interest in immunization regimens which induce liver-resident memory T cells as the parasite-infected liver is considered the primary site for protective T cell mediated immune responses which act to reduce liver-stage parasite burden (Fernandez-Ruiz et al., 2016; Gola et al., 2018; Olsen et al., 2018). It is possible that immune responses measured in the periphery or in splenocytes may not accurately reflect the vaccine-induced responses in the liver, so future studies should consider evaluation of liver-resident memory T cells in addition to the more traditional readouts from splenocytes.

Many VLP vaccine platforms have been developed, using structural proteins from various viral species. VLPs based on the murine polyomavirus are considered a promising VLP for vaccine development because of their reported humoral immunogenicity and their ability to be produced reproducibly with high yields (Chuan et al., 2008a; Liew et al., 2010, 2012; Middelberg et al., 2011; Anggraeni et al., 2013; Rivera-Hernandez et al., 2013; Wibowo et al., 2014; Seth et al., 2016; Tekewe et al., 2017). However, their capacity to induce robust antibody plus CD4^+^ and CD8^+^ T cell responses has not been thoroughly investigated. Accordingly, we evaluated both cellular and antibody responses using chimeric murine polyomavirus VLPs incorporating the CSP as a vaccine delivery platform, in the *P. yoelii* murine model.

It is well-established that VLPs are very effective at inducing antigen-specific antibody responses [reviewed in Grgacic and Anderson (2006)] likely due to their ability cross-link B cell surface receptors (Thönes et al., 2008) acting in a T cell independent manner (Snapper and Mond, 1993; Szomolanyi-Tsuda and Welsh, 1998). Consistent with that, herein we showed that our B cell VLP induced high IgG titres against the *Py*CSP B cell epitope. There was a significant decrease in antibody titers in the DNA prime/VLP boost group when the pooled VLP component was increased from 10 to 30 μg per VLP (Figure 5). This variation was not seen in the VLP only immunized mice, but those mice received three VLP doses and the additional doses may have normalized the responses.

We established that immunizations with either chimeric CD8 VLPs alone, or as pooled with a B cell VLP and CD4 T cell VLP were able to induce moderate levels of IFN-γ directed against the CD8^+^ T cell epitope presented as a synthetic peptide or in the context of whole antigen transfectant, with both ELISpot and ICS (Figures 3A,C, 4A,C). This antigen-specific recognition confirms that the CD8 chimeric VLP presented the incorporated CD8^+^ T cell epitope *in vivo* to naive CD8^+^ T cells, and that this presentation was via MHC class I molecules (Heath et al., 2004). These results are consistent with previous studies reporting that chimeric VLPs incorporating a single CD8^+^ T cell epitope from viral or ovalbumin targets induced CTL activity mediated by CD8^+^ T cells (Sedlik et al., 1997; Crisci et al., 2009). It is possible that our immunizations with pooled VLPs using 30 μg of each construct failed to protect mice from challenge as the induced CD8^+^ T cell response was insufficient to protect (Schmidt et al., 2008) with a mean frequency of 0.59% of CD8^+^ T cells expressing IFN-γ (Figure 4B).

We had anticipated that immunizing with a pool of VLPs which included the CD4 T cell peptide might result in an additive or synergistic response, as noted for immunization with a pool of the synthetic peptides. However, there was no evidence of any additive or synergistic effects on CD8^+^ T cell responses when VLPs were administered as a pool with the CD4 VLP; this was likely due to the very low responses induced by the CD4 VLP. The carrier MuPyV VP1 protein is likely to contain CD4^+^ T helper epitopes similar to those in the HBV core protein (Schödel et al., 1992; Bickert et al., 2007) and may provide carrier-related T cell help for antibody responses, but those would provide no antigen-specific help against challenge by the *Plasmodium* parasite. Poly (I:C) was selected to adjuvant the synthetic peptides *in vivo* because it is a known inducer of type I (Salem et al., 2005; Qiu and Cui, 2007; Coffman et al., 2010; Schneider-Ohrum et al., 2011) and II (Tewari et al., 2010; Kastenmüller et al., 2013) cellular responses. However, VLPs are generally considered self-adjuvating due to their viral structure (Stanley et al., 2006; Chackerian, 2007). There are however contradictory reports, with some reports showing that adjuvants are essential (Storni et al., 2002), beneficial (Freyschmidt et al., 2004; Ding et al., 2009) or not necessary (Mazeike et al., 2012; Rivera-Hernandez et al., 2013; Kawano et al., 2014). In our studies, we elected to exclude adjuvants so as to better understand the immunogenicity of our chimeric VLPs. We cannot however rule out the possibility that administration of the CD4 VLP with poly(I:C) could have enhanced the VLP-induced T cell response, but other data generated in our laboratory showed no enhancement of CD4^+^ T cell when pooled VLPs were co-administered with poly(I:C) (manuscript in preparation).

In our VLP constructs, each T cell epitope was inserted into surface-exposed regions of the VP1 protein, since we predicted that epitopes inserted into this region would be less likely to interfere with VLP formation. It is possible that this location may not have been optimal for the induction of cellular immune responses and that placing the epitopes on terminal regions may have been beneficial, as reported in one study in a chimeric calicivirus-like VLP system with ovalbumin CD8 epitopes showing significantly higher IFN-γ responses, target-specific lysis and protection (Crisci et al., 2009). It is also possible that the lack of responses to the CD4 epitope may have been a dose related issue; the CD4 peptide component of the 30 μg VLP dose was approximately 0.52 μg peptide epitope equivalent, whereas the peptide immunized mice received nearly 60 times the epitope per dose. Our immunogenicity assessments used lymphocytes taken from spleen and not draining lymph nodes, but the strong responses detected in splenocytes from the peptide-immunized mice would be expected to accurately reflect a response in the draining lymph nodes; if the VLPs induced a robust CD4^+^ T cell response then this should also have been detected in splenocytes.

To evaluate the protective capacity of our chimeric VLPs, we harvested livers 42 h post- sporozoite challenge, at the late liver-stage of the *P. yoelii* lifecycle before parasites were expected to release into the blood (Baer et al., 2007). This timepoint maximized the opportunity for VLP-induced protective immune responses to clear parasite-infected cells. Liver-stage protection has previously been achieved by adoptively transferring a $\text{CD}8_{280-288}^{+}$ T cell clone (Weiss et al., 1988), or by passive transfer of monoclonal antibodies specific to the *Py*CSP central repeat (Charoenvit et al., 1991). These protective antigenic epitopes were incorporated within our VLP chimeras, and generated epitope-specific CD8^+^ T cell and antibody responses. In our study, there was evidence that VLPs could induce protection against sporozoite challenge since sterile protection was seen in 2 of 5 mice and there was a reduction in parasite load; however, this protection was suboptimal. Our immunological data supports the induction of the type of immune response required to protect against *Plasmodium* sporozoite challenge, but the magnitude of those VLP-induced responses may have been insufficient to reach a protective threshold (Schmidt et al., 2010). This is consistent with other studies where protection correlated with the number of adoptively transferred clonal $\text{CD}8_{280-288}^{+}$ T cells where 20 × 10^6^ cells were required for sterile protection (Weiss et al., 1992), or with the number of memory CD8^+^ T cells stimulated by immunizations (Schmidt et al., 2008, 2010), or with the amount of passive transferred monoclonal antibodies (Charoenvit et al., 1991).

Indeed, we found that more robust antibody and cellular responses were induced by a prime/boost regimen as compared with homologous VLPs, and we anticipate that this would be further enhanced with the inclusion of a second VLP boost.

Overall, our data showed that the MuPyV chimeric VLP platform is capable of inducing antigen-specific antibody responses, and established for the first time for this vaccine platform, their capacity to induce CD8^+^ T cell immune responses. Future studies could evaluate alternative epitope insertion sites within the MuPyV VP1 protein to determine if other sites enhance cellular immune responses, or the inclusion of adjuvants in the vaccination regimen. Additional regions of the target antigens (e.g., repetitive regions targeted by antibody responses, together with T helper epitopes) could also be included. Alternative target antigens could be also evaluated since studies have suggested that multiple antigen targets may be required for protection from *Plasmodium* challenge (Doolan et al., 1996; John et al., 2005; Osier et al., 2008). With approximately 2,000 active genes, and 816 identified proteins present during *Plasmodium* liver-stage of infection (Tarun et al., 2008) part of the challenge is to identify the most optimal target antigens for induction of vaccine-induced protective immunity (Schussek et al., 2017).

## Data Availability

The datasets generated for this study can be obtained from the corresponding author upon reasonable request.

## Ethics Statement

All animal experiments were approved by the QIMR Berghofer Animal Ethics Committee and were conducted in accordance with the Australian Code of Practice for the Care and Use of Animals for Scientific Purposes (2004).

## Author Contributions

DP, SA, DD, and AM contributed conception and design of the study. DP, NW, YC, TR-H, LL, and AM contributed to construction of virus-like particles. DP, SA, and PG conducted mouse experiments. DP performed the statistical analysis. DP wrote the first draft of the manuscript. DD and SA edited the manuscript. All authors contributed to manuscript revision, read, and approved the submitted version.

### Conflict of Interest Statement

The University of Queensland (UQ) filed patents on the use of MuPyV as a vaccine platform. LL and AM contributed to those patents and, through their employment with UQ, hold an indirect interest in this intellectual property. The remaining authors declare that the research was conducted in the absence of any commercial or financial relationships that could be construed as a potential conflict of interest.

## References

[B1] Agnandji S. T. Lell B. Fernandes J. F. Abossolo B. P. Methogo B. G. Methogo B. G.. (2012). A phase 3 trial of RTS,S/AS01 malaria vaccine in African infants. N. Engl. J. Med. 367, 2284–2295. 10.1056/NEJMoa1208394 23136909 PMC10915853

[B2] Alonso P. L. Brown G. Arevalo-Herrera M. Binka F. Chitnis C. Collins F. (2011). A research agenda for malaria eradication: vaccines. PLoS Med. 8:e1000398 10.1371/journal.pmed.1000406 21311586 PMC3026701

[B3] Anggraeni M. R. Connors N. K. Wu Y. Chuan Y. P. Lua L. H. Middelberg A. P. (2013). Sensitivity of immune response quality to influenza helix 190 antigen structure displayed on a modular virus-like particle. Vaccine 31, 4428–4435. 10.1016/j.vaccine.2013.06.087 23845811

[B4] Baer K. Klotz C. Kappe S. H. Schnieder T. Frevert U. (2007). Release of hepatic *Plasmodium yoelii* merozoites into the pulmonary microvasculature. PLoS Pathog. 3:e171. 10.1371/journal.ppat.0030171 17997605 PMC2065874

[B5] Bickert T. Wohlleben G. Brinkman M. Trujillo-Vargas C. M. Ruehland C. Reiser C. O.. (2007). Murine polyomavirus-like particles induce maturation of bone marrow-derived dendritic cells and proliferation of T cells. Med. Microbiol. Immunol. 196, 31–39. 10.1007/s00430-006-0026-x 16917781

[B6] Chackerian B. (2007). Virus-like particles: flexible platforms for vaccine development. Expert Rev Vaccines 6, 381–390. 10.1586/14760584.6.3.381 17542753

[B7] Chackerian B. Lenz P. Lowy D. R. Schiller J. T. (2002). Determinants of autoantibody induction by conjugated papillomavirus virus-like particles. J. Immunol. 169, 6120–6126. 10.4049/jimmunol.169.11.6120 12444114

[B8] Charoenvit Y. Mellouk S. Cole C. Bechara R. Leef M. F. Sedegah M. (1991). Monoclonal, but not polyclonal, antibodies protect against Plasmodium-yoelii sporozoites. J. Immunol. 146, 1020–1025. 1988490

[B9] Chuan Y. P. Fan Y. Y. Lua L. Middelberg A. P. (2008b). Quantitative analysis of virus-like particle size and distribution by field-flow fractionation. Biotechnol. Bioeng. 99, 1425–1433. 10.1002/bit.21710 18023039

[B10] Chuan Y. P. Lua L. H. Middelberg A. P. Chuan Y. P. Lua L. H. Middelberg A. P. (2008a). High-level expression of soluble viral structural protein in *Escherichia coli*. J. Biotechnol. 134, 64–71. 10.1016/j.jbiotec.2007.12.004 18249455

[B11] Coffman R. L. Sher A. Seder R. A. (2010). Vaccine adjuvants: putting innate immunity to work. Immunity. 33, 492–503. 10.1016/j.immuni.2010.10.002 21029960 PMC3420356

[B12] Collins K. A. Snaith R. Cottingham M. G. Gilbert S. C. Hill A. V. S. (2017). Enhancing protective immunity to malaria with a highly immunogenic virus-like particle vaccine. Sci. Rep. 7:46621. 10.1038/srep46621 28422178 PMC5395940

[B13] Crisci E. Almanza H. Mena I. Córdoba L. Gómez-Casado E. Castón J. R.. (2009). Chimeric calicivirus-like particles elicit protective anti-viral cytotoxic responses without adjuvant. Virology 387, 303–312. 10.1016/j.virol.2009.02.045 19327809

[B14] Ding F. X. Wang F. Lu Y. M. Li K. Wang K. H. He X. W.. (2009). Multiepitope peptide-loaded virus-like particles as a vaccine against hepatitis B virus-related hepatocellular carcinoma. Hepatology 49, 1492–1502. 10.1002/hep.22816 19206147

[B15] Doolan D. L. Hoffman S. L. (2000). The complexity of protective immunity against liver-stage malaria. J. Immunol. 165, 1453–1462. 10.4049/jimmunol.165.3.1453 10903750

[B16] Doolan D. L. Sedegah M. Hedstrom R. C. Hobart P. Charoenvit Y. Hoffman S. L. (1996). Circumventing genetic restriction of protection against malaria with multigene DNA immunization: CD8(+) T cell-, interferon gamma-, and nitric oxide-dependent immunity. J. Exp. Med. 183, 1739–1746. 10.1084/jem.183.4.1739 8666931 PMC2192484

[B17] Draper S. J. Angov E. Horii T. Miller L. H. Srinivasan P. Theisen M.. (2015). Recent advances in recombinant protein-based malaria vaccines. Vaccine 33, 7433–7443. 10.1016/j.vaccine.2015.09.093 26458807 PMC4687528

[B18] Fernandez-Ruiz D. Ng W. Y. Holz L. E. Ma J. Z. Zaid A. Wong Y. C.. (2016). Liver-resident memory CD8(+) T cells form a front-line defense against malaria liver-stage infection. Immunity 45, 889–902. 10.1016/j.immuni.2016.08.011 27692609

[B19] Franke E. D. Corradin G. Hoffman S. L. (1997). Induction of protective CTL responses against the *Plasmodium yoelii* circumsporozoite protein by immunization with peptides. J. Immunol. 159, 3424–3433. 9317141

[B20] Franke E. D. Sette A. Sacci J. Southwood S. Corradin G. Hoffman S. L. (2000). A subdominant CD8(+) cytotoxic T lymphocyte (CTL) epitope from the *Plasmodium yoelii* circumsporozoite protein induces CTLs that eliminate infected hepatocytes from culture. Infect. Immun. 68, 3403–3411. 10.1128/IAI.68.6.3403-3411.2000 10816491 PMC97612

[B21] Freyschmidt E. J. Alonso A. Hartmann G. Gissmann L. (2004). Activation of dendritic cells and induction of T cell responses by HPV 16 L1/E7 chimeric virus-like particles are enhanced by CpG ODN or sorbitol. Antivir. Ther. 9, 479–489. 15456078

[B22] Gola A. Silman D. Walters A. A. Sridhar S. Uderhardt S. Salman A. M.. (2018). Prime and target immunization protects against liver-stage malaria in mice. Sci. Transl. Med. 10:460. 10.1126/scitranslmed.aap9128 30257955

[B23] Gordon D. M. McGovern T. W. Krzych U. Cohen J. C. Schneider I. LaChance R.. (1995). Safety, immunogenicity, and efficacy of a recombinantly produced Plasmodium-falciparum circumsporozoite-protein hepatitis-b surface-antigen subunit vaccine. J. Infect. Dis. 171, 1576–1585. 10.1093/infdis/171.6.1576 7769295

[B24] Grgacic E. V. Anderson D. A. (2006). Virus-like particles: passport to immune recognition. Methods 40, 60–65. 10.1016/j.ymeth.2006.07.018 16997714 PMC7128828

[B25] Grillot D. Michel M. Müller I. Tougne C. Rènia L. Mazier D.. (1990). Immune responses to defined epitopes of the circumsporozoite protein of the murine malaria parasite, *Plasmodium yoelii*. Eur. J. Immunol. 20, 1215–1222. 10.1002/eji.1830200604 1695152

[B26] Heath W. R. Belz G. T. Behrens G. M. Smith C. M. Forehan S. P. Parish I. A.. (2004). Cross-presentation, dendritic cell subsets, and the generation of immunity to cellular antigens. Immunol. Rev. 199, 9–26. 10.1111/j.0105-2896.2004.00142.x 15233723

[B27] Janitzek C. M. Matondo S. Thrane S. Nielsen M. A. Kavishe R. Mwakalinga S. B.. (2016). Bacterial superglue generates a full-length circumsporozoite protein virus-like particle vaccine capable of inducing high and durable antibody responses. Malar. J. 15:545. 10.1186/s12936-016-1574-1 27825348 PMC5101663

[B28] John C. C. Moormann A. M. Pregibon D. C. Sumba P. O. McHugh M. M. Narum D. L.. (2005). Correlation of high levels of antibodies to multiple pre-erythrocytic *Plasmodium falciparum* antigens and protection from infection. Am. J. Trop. Med. Hyg. 73, 222–228. 10.4269/ajtmh.2005.73.222 16014863

[B29] Kastenmüller K. Espinosa D. A. Trager L. Stoyanov C. Salazar A. M. Pokalwar S.. (2013). Full-length *Plasmodium falciparum* circumsporozoite protein administered with long-chain poly (I.C) or the Toll-like receptor 4 agonist glucopyranosyl lipid adjuvant-stable emulsion elicits potent antibody and CD4+ T cell immunity and protection in mice. Infect. Immun. 81, 789–800. 10.1128/IAI.01108-12 23275094 PMC3584875

[B30] Kawano M. Morikawa K. Suda T. Ohno N. Matsushita S. Akatsuka T.. (2014). Chimeric SV40 virus-like particles induce specific cytotoxicity and protective immunity against influenza A virus without the need of adjuvants. Virology 448, 159–167. 10.1016/j.virol.2013.10.010 24314646

[B31] Kogot J. M. Sarkes D. A. Val-Addo I. Pellegrino P. M. Stratis-Cullum D. N. (2012). Increased affinity and solubility of peptides used for direct peptide ELISA on polystyrene surfaces through fusion with a polystyrene-binding peptide tag. Biotechniques 52, 95–102. 10.2144/000113810 22313407

[B32] Kumada Y. Kuroki D. Yasui H. Ohse T. Kishimoto M. (2010). Characterization of polystyrene-binding peptides (PS-tags) for site-specific immobilization of proteins. J. Biosci. Bioeng. 109, 583–587. 10.1016/j.jbiosc.2009.11.005 20471598

[B33] Kushnir N. Streatfield S. J. Yusibov V. (2012). Virus-like particles as a highly efficient vaccine platform: diversity of targets and production systems and advances in clinical development. Vaccine 31, 58–83. 10.1016/j.vaccine.2012.10.083 23142589 PMC7115575

[B34] Liew M. W. Rajendran A. Middelberg A. P. (2010). Microbial production of virus-like particle vaccine protein at gram-per-litre levels. J. Biotechnol. 150, 224–231. 10.1016/j.jbiotec.2010.08.010 20797415

[B35] Liew M. W. O. Chuan Y. P. Middelberg A. P. J. (2012). High-yield and scalable cell-free assembly of virus-like particles by dilution. Biochem. Eng. J. 67, 88–96. 10.1016/j.bej.2012.05.007

[B36] Lipin D. I. Lua L. H. Middelberg A. P. (2008). Quaternary size distribution of soluble aggregates of glutathione-S-transferase-purified viral protein as determined by asymmetrical flow field flow fractionation and dynamic light scattering. J. Chromatogr. A 1190, 204–214. 10.1016/j.chroma.2008.03.032 18395215

[B37] Majarian W. R. Daly T. M. Weidanz W. P. Long C. A. (1984). Passive immunization against murine malaria with an IgG3 monoclonal antibody. J. Immunol. 132, 3131–3137. 6725950

[B38] Mazeike E. Gedvilaite A. Blohm U. (2012). Induction of insert-specific immune response in mice by hamster polyomavirus VP1 derived virus-like particles carrying LCMV GP33 CTL epitope. Virus Res. 163, 2–10. 10.1016/j.virusres.2011.08.003 21864590 PMC7114473

[B39] Mettens P. Dubois P. M. Demoitié M. A. Bayat B. Donner M. N. Bourguignon P.. (2008). Improved T cell responses to *Plasmodium falciparum* circumsporozoite protein in mice and monkeys induced by a novel formulation of RTS,S vaccine antigen. Vaccine 26, 1072–1082. 10.1016/j.vaccine.2007.12.018 18258343

[B40] Middelberg A. P. Rivera-Hernandez T. Wibowo N. Lua L. H. Fan Y. Magor G.. (2011). A microbial platform for rapid and low-cost virus-like particle and capsomere vaccines. Vaccine 29, 7154–7162. 10.1016/j.vaccine.2011.05.075 21651936

[B41] Mishra S. Nussenzweig R. S. Nussenzweig V. (2012). Antibodies to Plasmodium circumsporozoite protein (CSP) inhibit sporozoite's cell traversal activity. J. Immunol. Methods. 377, 47–52. 10.1016/j.jim.2012.01.009 22306356 PMC3310221

[B42] Noe A. R. Espinosa D. Li X. Coelho-Dos-Reis J. G. Funakoshi R. Giardina S.. (2014). A full-length *Plasmodium falciparum* recombinant circumsporozoite protein expressed by *Pseudomonas fluorescens* platform as a malaria vaccine candidate. PLoS ONE 9:e107764. 10.1371/journal.pone.0107764 25247295 PMC4172688

[B43] Ohlschläger P. Osen W. Dell K. Faath S. Garcea R. L. Jochmus I.. (2003). Human papillomavirus type 16 L1 capsomeres induce L1-specific cytotoxic T lymphocytes and tumor regression in C57BL/6 mice. J. Virol. 77, 4635–4645. 10.1128/JVI.77.8.4635-4645.2003 12663770 PMC152157

[B44] Olotu A. Fegan G. Wambua J. Nyangweso G. Leach A. Lievens M.. (2016). Seven-year efficacy of RTS,S/AS01 malaria vaccine among young african children. N. Engl. J. Med. 374, 2519–2529. 10.1056/NEJMoa1515257 27355532 PMC4962898

[B45] Olsen T. M. Stone B. C. Chuenchob V. Murphy S. C. (2018). Prime-and-trap malaria vaccination to generate protective CD8(+) liver-resident memory T cells. J. Immunol. 201, 1984–1993. 10.4049/jimmunol.1800740 30127085

[B46] Osier F. H. Fegan G. Polley S. D. Murungi L. Verra F. Tetteh K. K.. (2008). Breadth and magnitude of antibody responses to multiple *Plasmodium falciparum* merozoite antigens are associated with protection from clinical malaria. Infect. Immun. 76, 2240–2248. 10.1128/IAI.01585-07 18316390 PMC2346713

[B47] Qiu F. Cui Z. (2007). CD4+ T helper cell response is required for memory in CD8+ T lymphocytes induced by a poly(I:C)-adjuvanted MHC I-restricted peptide epitope. J. Immunother. 30, 180–189. 10.1097/01.cji.0000211330.61019.6f 17471165

[B48] Rivera-Hernandez T. Hartas J. Wu Y. Chuan Y. P. Lua L. H. Good M.. (2013). Self-adjuvanting modular virus-like particles for mucosal vaccination against group A *streptococcus* (GAS). Vaccine 31, 1950–1955. 10.1016/j.vaccine.2013.02.013 23422147

[B49] Salem M. L. Kadima A. N. Cole D. J. Gillanders W. E. (2005). Defining the antigen-specific T-Cell response to vaccination and poly (I:C)/TLR3 signaling - evidence of enhanced primary and memory CD8 T-Cell responses and antitumor immunity. J. Immunother. 28, 220–228. 10.1097/01.cji.0000156828.75196.0d 15838378

[B50] Salunke D. M. Caspar D. L. Garcea R. L. (1986). Self-assembly of purified polyomavirus capsid protein VP1. Cell 46, 895–904. 10.1016/0092-8674(86)90071-1 3019556

[B51] Schmidt N. W. Butler N. S. Badovinac V. P. Harty J. T. (2010). Extreme CD8 T cell requirements for anti-malarial liver-stage immunity following immunization with radiation attenuated sporozoites. PLoS Pathog. 6:e1000998. 10.1371/journal.ppat.1000998 20657824 PMC2904779

[B52] Schmidt N. W. Podyminogin R. L. Butler N. S. Badovinac V. P. Tucker B. J. Bahjat K. S.. (2008). Memory CD8 T cell responses exceeding a large but definable threshold provide long-term immunity to malaria. Proc. Natl. Acad. Sci. U.S.A. 105, 14017–14022. 10.1073/pnas.0805452105 18780790 PMC2544571

[B53] Schneider-Ohrum K. Giles B. M. Weirback H. K. Williams B. L. DeAlmeida D. R. Ross T. M. (2011). Adjuvants that stimulate TLR3 or NLPR3 pathways enhance the efficiency of influenza virus-like particle vaccines in aged mice. Vaccine 29, 9081–9092. 10.1016/j.vaccine.2011.09.051 21963872 PMC6690196

[B54] Schödel F. Moriarty A. M. Peterson D. L. Zheng J. A. Hughes J. L. Will H.. (1992). The position of heterologous epitopes inserted in hepatitis-B Virus core particles determines their immunogenicity. J. Virol. 66, 106–114. 1370083 10.1128/jvi.66.1.106-114.1992 PMC238265

[B55] Schussek S. Groves P. L. Apte S. H. Doolan D. L. (2013). Highly sensitive quantitative real-time PCR for the detection of Plasmodium liver-stage parasite burden following low-dose sporozoite challenge. PLoS ONE 8:e77811. 10.1371/journal.pone.0077811 24098596 PMC3788780

[B56] Schussek S. Trieu A. Apte S. H. Sidney J. Sette A. Doolan D. L. (2017). Novel *Plasmodium antigens* identified via genome-based antibody screen induce protection associated with polyfunctional T cell responses. Sci. Rep. 7:18. 10.1038/s41598-017-15354-0 29118376 PMC5678182

[B57] Sedegah M. Hedstrom R. Hobart P. Hoffman S. L. (1994). Protection against malaria by immunization with plasmid DNA encoding circumsporozoite protein. Proc. Natl. Acad. Sci. U.S.A. 91, 9866–9870. 10.1073/pnas.91.21.9866 7937907 PMC44918

[B58] Seder R. A. Hill A. V. (2000). Vaccines against intracellular infections requiring cellular immunity. Nature 406, 793–798. 10.1038/35021239 10963610

[B59] Sedlik C. Saron M. Sarraseca J. Casal I. Leclerc C. (1997). Recombinant parvovirus-like particles as an antigen carrier: a novel nonreplicative exogenous antigen to elicit protective antiviral cytotoxic T cells. Proc. Natl. Acad. Sci. U.S.A. 94, 7503–7508. 10.1073/pnas.94.14.7503 9207121 PMC23851

[B60] Seth A. Kong I. G. Lee S. H. Yang J. Y. Lee Y. S. Kim Y.. (2016). Modular virus-like particles for sublingual vaccination against group A *streptococcus*. Vaccine 34, 6472–6480. 10.1016/j.vaccine.2016.11.008 27866769

[B61] Snapper C. M. Mond J. J. (1993). Towards a comprehensive view of immunoglobulin class switching. Immunol. Today 14, 15–17. 10.1016/0167-5699(93)90318-F 8442856

[B62] Stanley M. Lowy D. R. Frazer I. (2006). Chapter 12: prophylactic HPV vaccines: underlying mechanisms. Vaccine 24(Suppl 3), 106–113. 10.1016/j.vaccine.2006.05.110 16949996

[B63] Stanley M. A. (2006). Human papillomavirus vaccines. Rev. Med. Virol. 16, 139–149. 10.1002/rmv.498 16710836

[B64] Storni T. Lechner F. Erdmann I. Bächi T. Jegerlehner A. Dumrese T.. (2002). Critical role for activation of antigen-presenting cells in priming of cytotoxic T cell responses after vaccination with virus-like particles. J. Immunol. 168, 2880–2886. 10.4049/jimmunol.168.6.2880 11884458

[B65] Szomolanyi-Tsuda E. Welsh R. M. (1998). T-cell-independent antiviral antibody responses. Curr. Opin. Immunol. 10, 431–435. 10.1016/S0952-7915(98)80117-9 9722919

[B66] Tamminga C. Sedegah M. Regis D. Chuang I. Epstein J. E. Spring M.. (2011). Adenovirus-5-vectored *P. falciparum* vaccine expressing CSP and AMA1. Part B: safety, immunogenicity and protective efficacy of the CSP component. PLoS ONE 6:e25868. 10.1371/journal.pone.0025868 22003411 PMC3189219

[B67] Tarun A. S. Peng X. Dumpit R. F. Ogata Y. Silva-Rivera H. Camargo N.. (2008). A combined transcriptome and proteome survey of malaria parasite liver stages. Proc. Natl. Acad. Sci. U.S.A. 105, 305–310. 10.1073/pnas.0710780104 18172196 PMC2224207

[B68] Tegerstedt K. Lindencrona J. A. Curcio C. Andreasson K. Tullus C. Forni G.. (2005). A single vaccination with polyomavirus VP1/VP2Her2 virus-like particles prevents outgrowth of HER-2/neu-expressing tumors. Cancer Res. 65, 5953–5957. 10.1158/0008-5472.CAN-05-0335 15994974

[B69] Tekewe A. Fan Y. Tan E. Middelberg A. P. Lua L. H. (2017). Integrated molecular and bioprocess engineering for bacterially produced immunogenic modular virus-like particle vaccine displaying 18 kDa rotavirus antigen. Biotechnol. Bioeng. 114, 397–406. 10.1002/bit.26068 27497268

[B70] Tewari K. Flynn B. J. Boscardin S. B. Kastenmueller K. Salazar A. M. Anderson C. A.. (2010). Poly(I:C) is an effective adjuvant for antibody and multi-functional CD4+ T cell responses to *Plasmodium falciparum* circumsporozoite protein (CSP) and alphaDEC-CSP in non human primates. Vaccine 28, 7256–7266. 10.1016/j.vaccine.2010.08.098 20846528 PMC3004225

[B71] Thönes N. Herreiner A. Schädlich L. Piuko K. Müller M. (2008). A direct comparison of human papillomavirus type 16 L1 particles reveals a lower immunogenicity of capsomeres than viruslike particles with respect to the induced antibody response. J. Virol. 82, 5472–5485. 10.1128/JVI.02482-07 18385253 PMC2395182

[B72] Tinto H. D'alessandro U. Sorgho H. Valea I. Tahita M. C. Kabore W.. (2015). Efficacy and safety of RTS,S/AS01 malaria vaccine with or without a booster dose in infants and children in Africa: final results of a phase 3, individually randomised, controlled trial. Lancet 386, 31–45. 10.1016/S0140-6736(15)60721-8 25913272 PMC5626001

[B73] Walsh D. S. Gettayacamin M. Leitner W. W. Lyon J. A. Stewart V. A. Marit G.. (2006). Heterologous prime-boost immunization in rhesus macaques by two, optimally spaced particle-mediated epidermal deliveries of *Plasmodium falciparum* circumsporozoite protein-encoding DNA, followed by intramuscular RTS,S/AS02A. Vaccine 24, 4167–4178. 10.1016/j.vaccine.2006.02.041 16574282

[B74] Weiss W. R. Berzofsky J. A. Houghten R. A. Sedegah M. Hollindale M. Hoffman S. L. (1992). A T-cell clone directed at the circumsporozoite protein which protects mice against both Plasmodium-yoelii and Plasmodium-berghei. J. Immunol. 149, 2103–2109. 1517574

[B75] Weiss W. R. Mellouk S. Houghten R. A. Sedegah M. Kumar S. Good M. F.. (1990). Cytotoxic T cells recognize a peptide from the circumsporozoite protein on malaria-infected hepatocytes. J. Exp. Med. 171, 763–773. 10.1084/jem.171.3.763 1689762 PMC2187765

[B76] Weiss W. R. Sedegah M. Beaudoin R. L. Miller L. H. Good M. F. (1988). CD8+ T cells (cytotoxic/suppressors) are required for protection in mice immunized with malaria sporozoites. Proc. Natl. Acad. Sci. U.S.A. 85, 573–576. 10.1073/pnas.85.2.573 2963334 PMC279593

[B77] Weiss W. R. Sedegah M. Berzofsky J. A. Hoffman S. L. (1993). The role of CD4+ T cells in immunity to malaria sporozoites. J. Immunol. 151, 2690–2698. 8103069

[B78] Wibowo N. Hughes F. K. Fairmaid E. J. Lua L. H. Brown L. E. Middelberg A. P. (2014). Protective efficacy of a bacterially produced modular capsomere presenting M2e from influenza: extending the potential of broadly cross-protecting epitopes. Vaccine 32, 3651–3655. 10.1016/j.vaccine.2014.04.062 24795225

[B79] World Malaria Report (2017). World Health Organization. Available online at: http://www.who.int/malaria/publications/world-malaria-report-2017/en/ (accessed January 25, 2019).

[B80] Yoshida K. Iyori M. Blagborough A. M. Salman A. M. Dulal P. Sala K. A.. (2018). Adenovirus-prime and baculovirus-boost heterologous immunization achieves sterile protection against malaria sporozoite challenge in a murine model. Sci. Rep. 8:3896. 10.1038/s41598-018-21369-y 29497047 PMC5832798

